# *Solotvynia*, a New Coccoid Lineage among the Ulvophyceae (Chlorophyta)

**DOI:** 10.3390/microorganisms12050868

**Published:** 2024-04-26

**Authors:** Tatyana Darienko, Thomas Pröschold

**Affiliations:** 1 Department of Applied Bioinformatics, Institute for Microbiology and Genetics, Georg-August-University of Göttingen, D-37077 Göttingen, Germany; tdarien@gwdg.de; 2 Research Department for Limnology, Leopold-Franzens-University of Innsbruck, A-5310 Mondsee, Austria

**Keywords:** *Chlorocystis*, distribution, *Halochlorococcum*, *Ignatius*, ITS-2 secondary structure, marine coccoids, SSU/ITS phylogeny, *Symbiochlorum*

## Abstract

Coccoid Ulvophyceae are often overlooked despite their wide distribution. They occur as epiphytes on marine seaweeds or grow on stones or on shells of mussels and corals. Most of the species are not easy to identify based solely on morphology. However, they form two groups based on the flagellated cells during asexual reproduction. The biflagellated coccoids are monophyletic and represent the genus *Sykidion* (Sykidiales). In contrast, the quadriflagellated taxa are polyphyletic and belong to different genera and orders. The newly investigated strains NIES-1838 and NIES-1839, originally identified as *Halochlorococcum,* belong to the genus *Chlorocystis* (*C. john-westii*) among the order Chlorocystidales. The unidentified strain CCMP 1293 had almost an identical SSU and ITS-2 sequence to *Symbiochlorum hainanense* (Ignatiales) but showed morphological differences (single chloroplast, quadriflagellated zoospores) compared with the original description of this species (multiple chloroplasts, aplanospores). Surprisingly, the strain SAG 2662 (= ULVO-129), together with the published sequence of MBIC 10461, formed a new monophyletic lineage among the Ulvophyceae, which is highly supported in all of the bootstrap and Bayesian analyses and approximately unbiased tests of user-defined trees. This strain is characterized by a spherical morphology and also form quadriflagellated zoospores, have a unique ITS-2 barcode, and can tolerate a high variation of salinities. Considering our results, we emend the diagnosis of *Symbiochlorum* and propose the new genus *Solotvynia* among the new order Solotvyniales.

## 1. Introduction

Benthic marine coccoid green algae are often overlooked and are rarely studied in detail despite them being known about for a long time. Most of these algae occur as epi/endophytes on or in different seaweeds or grow on the shells of mussels or corals. Therefore, these green algae were classified as being members of the genus *Chlorochytrium*, which was established by Cohn [1] for a coccoid green alga growing inside the leaves of different *Lemna* species. Bristol [2] reviewed the taxonomic status of freshwater and marine species belonging to this genus. Her review contains all of the described taxa and recognized 13 species. However, Wright [3] and Reinhard [4] had already demonstrated that marine coccoid green algae differ from those of the freshwater genus, and therefore described the genera *Sykidion* and *Chlorocystis*, respectively. Since then, many new taxa have been established based on microscopical studies of collected field material. Moewus [5] found a coccoid green alga as an endophyte of an unidentified *Chaetophora* species and named it *Symbiosphaera marina*. This species is characterized by reticulated chloroplasts and quadriflagellated zoospores. Unfortunately, no cultured material of this species is available. Therefore, the taxonomic status of *Symbiosphaera* remains unresolved, which has already been mentioned by Kornmann and Sahling [6], who emended the description of *Chlorocystis cohnii* (Wright) Reinhard. In addition, they established several new species of the genus *Halochlorococcum*, which was originally described by Dangeard [7]. The latter genus was formally incorrectly described, and therefore all of the other species were also invalid according to the International Code for Nomenclature (ICN). However, all of these species were later validated by Guiry [8]. Fortunately, several species described by Kornmann and Sahling [6] are available in public culture collections and have been taxonomically revised by Darienko et al. [9] using morphological and molecular methods. All these species (except for the species *H. marinum*) were transferred to the genus *Chlorocystis*. Morphologically, all of these species are difficult to identify because they show a high phenotypic plasticity, especially if they are grown under different salinities. A common feature is the production of quadriflagellated zoospores during asexual reproduction. Kornmann and Sahling [6] recognized in *Chlorocystis cohnii* a “*Codiolum*” stage in its life cycle in which it was a unicellular sporophyte, and, therefore, excluded this genus from the chlorophyte order Chlorococcales. They described a new order of Chlorocystidales among their Codiolophyceae (= Ulvophyceae s.str.). This order was emended by adding the sarcinoid genus *Desmochloris* identified by Watanabe, Kuroda, and Maiwa [9]. Among the Ulvophyceae, other lineages containing coccoid members were established. The soil alga *Ignatius tetrasporus* described by Bold and MacEntree [10] represents its own lineage within the Ulvophyceae [11] and was described as Ignatiales by Skaloud et al. [12]. This alga also reproduces asexually through quadriflagellated zoospores and has a reticulated chloroplast. Interestingly, among this order, Gong et al. [13] found another coccoid green alga (*Symbiochlorum*) isolated from bleached corals, which is characterized by the presence of aplanospores during asexual reproduction. Darienko et al. [9] proposed the additional order Sykidiales among the Ulvophyceae based on marine coccoids that reproduced by biflagellated zoospores. The genus *Sykidion,* originally described by Wright [3], contains three species, *S. dyeri* and *S. droebakense* described by Wille [14], and *S. marina*, a species originally described as *Pseudoneochloris* by Watanabe et al. [15].

The class Ulvophyceae is one of the major lineages of the Chlorophyta, which is characterized by the counterclockwise orientation of the basal bodies in the ultrastructure of flagellated cells [16]. Currently, this class contains the following orders: Oltmannsiellopsidales, Chlorocystidales, Ulvales, Sykidiales, Ulotrichales, Acrosiphoniales, Ignatiales, and Scotinosphaerales. Leliaert et al. [17] demonstrated that the orders Bryopsidales, Trentepohliales, Cladophorales, and Dasycladales, also originally identified as Ulvophyceae, represent their own lineages among the Chlorophyta, which van den Hoek et al. [18] has already named at the class level.

This study aimed to clarify the phylogenetic position of new marine coccoid green algae that reproduce by quadriflagellated zoospores. An integrative approach (combination of the morphological data with molecular phylogenetic results including genetic synapomorphies) revealed that all investigated strains belong to different lineages of the Ulvophyceae. Considering these results in combination with detailed investigations of the morphology and reproduction of these species, we emended the diagnosis of the genus *Symbiochlorum* and proposed a new genus and order of the Ulvophyceae: *Solotvynia,* Solotvyniales.

## 2. Materials and Methods

### 2.1. Cultures and Light Microscopy

The following strains were investigated:

The strain ULVO-129 was isolated from a water sample taken from a hypersaline artificial lake in Solotvyno, Ukraine (47°57′20″ N, 23°52′16″ E). These salt lakes are located near the Ukrainian–Romanian border, which has been the result of salt mining since the 13th century. The salt pits were then filled with groundwater. The salinity of these lakes varies up to more than 100 psu. This strain has been deposited in the Culture Collection of Algae, University of Göttingen, Germany (SAG; http://sagdb.uni-goettingen.de) and is available under the number SAG 2662.

The strain CCMP 1293, obtained from the Provasoli-Guillard National Center for Culture of Marine Phytoplankton, Bigelow, Maine, USA (NCMA formerly CCMP; https://ncma.bigelow.org), was isolated from a squeezed sample of *Lissoclinum patella*, which was collected from Palau Island (7°32′25″ N, 134°35′21″ E)

The strains NIES-1838 and NIES-1839, obtained in the Microbial Culture Collection at the National Institute for Environmental Studies, Tsukuba, Japan (NIES; https://mcc.nies.go.jp), were isolated from marine water samples collected from Yumenoshima, Tokyo, Japan (35°39′00″ N, 139°49′55″ E).

The algae were grown in Seawater Medium (SWES, medium 5 in Schlösser [19]), and modified Artificial Seawater Medium (MASM, medium 25 in Schlösser [20]; modified by adding 30 mL soil extract per liter). In addition, the strain SAG 2662 was cultivated on Basal medium (ES, medium 1 in Schlösser [19]), brackish medium (1/2SWES, medium 6 in Schlösser [19]), and *Dunaliella* medium (medium 14 in Schlösser [19]) to observe the phenotypic plasticity. The experiment was conducted as follows: A good-growing starter culture on SWES medium was either transferred to 1/2SWES or *Dunaliella* media. After growth, the 1/2SWES culture was used for inoculation on the ES medium. After the adaptation phase, all inoculated cultures showed good growth. Long-term experiments were not conducted. All species were cultivated in small petri dishes or test tubes with 10 mL agar medium (1.5% *w*/*v*) at 20 °C at light intensity of 20 µE/m^2^s, and a light/dark cycle of 14:10 h. The light microscopical observations were conducted after a growth of four weeks. The mature vegetative cells were investigated near the end of the light period. For the documentation of the micrographs, an Olympus BX-60 microscope (Olympus, Tokyo, Japan) equipped with a Prog Res C14 plus camera and the Prog Res Capture Pro imaging system (version 2.9.0.1), both from Jenoptik, Jena, Germany) were used.

### 2.2. DNA Extraction, PCR, Sequencing, and Phylogenetic Analyses

**Figure 1:**
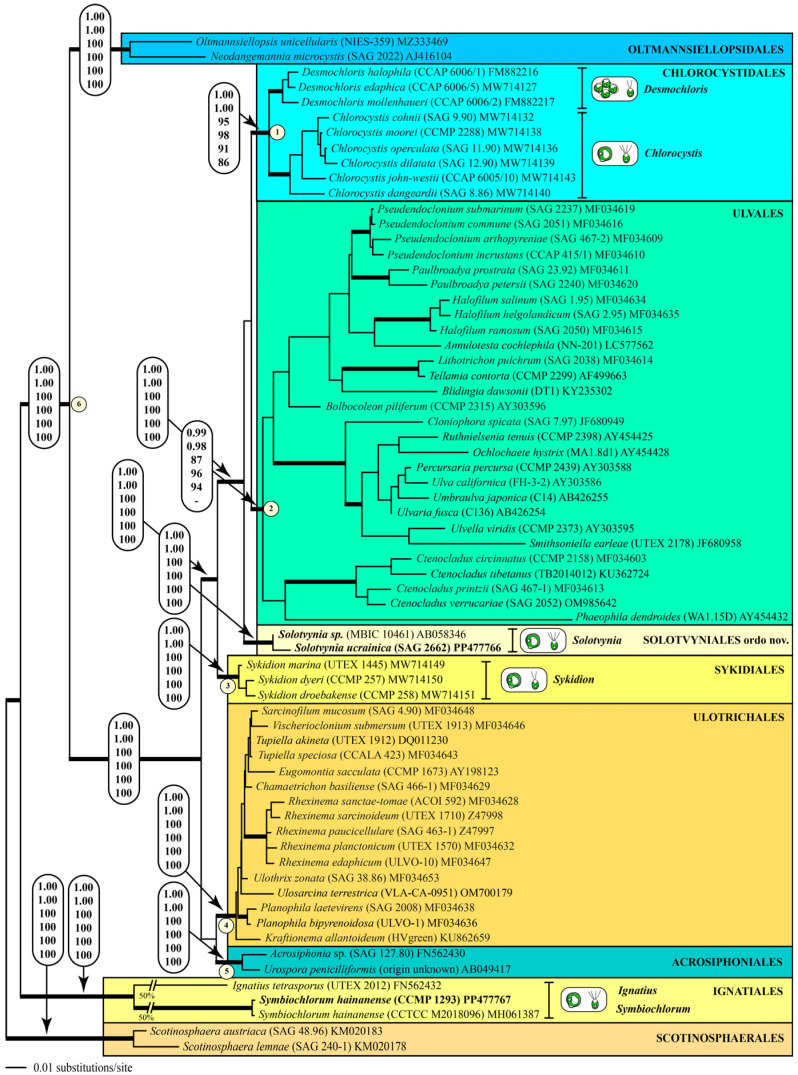
Molecular phylogeny of the Ulvophyceae based on SSU rDNA sequence comparisons. The phylogenetic tree shown was constructed using the maximum likelihood method based on a data set of 1780 aligned positions of 67 taxa using PAUP 4.0a (build169). For the analysis, the GTR+I+G (base frequencies: A 0.24763, C 0.21994, G 0.27379, U 0.25864; rate matrix A–C 1.1859, A–G 2.3460, A–U 1.4208, C–G 0.8191, C–U 4.4100, G–U 1.0000) with the proportion of invariable sites (I = 0.5216) and gamma shape parameter (G = 0.4680) was chosen, which was calculated as the best model using the automated model selection tool implemented in PAUP. The branches in bold are highly supported by all of the analyses (Bayesian values > 0.95 calculated with PHASE and MrBayes; bootstrap values > 70% calculated with PAUP using maximum likelihood, neighbor-joining, maximum parsimony, and RAxML using maximum likelihood). The sister group Scotinosphaerales was chosen as an outgroup. The clade designations follow the currently accepted order classification of the Ulvophyceae. The newly sequenced strains are highlighted in bold.

**Figure 2:**
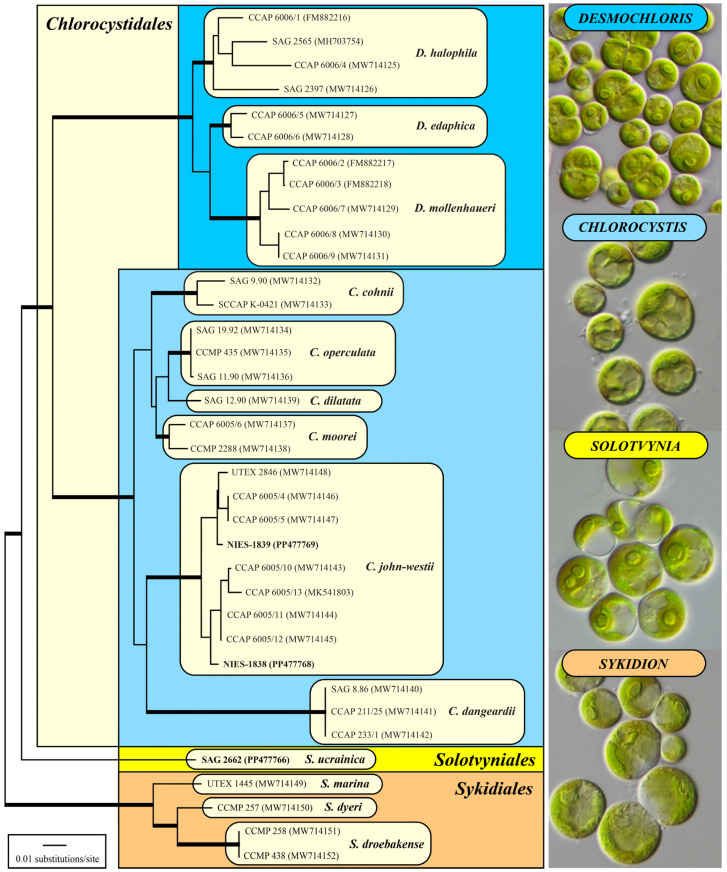
Molecular phylogeny of the coccoid ulvophytes based on SSU and ITS rDNA sequence comparisons. The phylogenetic trees shown were constructed using the maximum likelihood method based on the data sets (2389 aligned positions of 36 taxa) using PAUP 4.0a (build169). For the analyses, the best model was calculated by the automated model selection tool implemented in PAUP. The setting of the best model was given as follows: SYM+I+G (base frequencies: equal; rate matrix A–C 1.1884, A–G 2.2434, A–U 1.6291, C–G 1.1783, C–U 4.3437, G–U 1.0000) with the proportion of invariable sites (I = 0.7046) and gamma shape parameter (G = 0.4966). The branches in bold are highly supported by all of the analyses (Bayesian values > 0.95 calculated with PHASE and MrBayes; bootstrap values > 70% calculated with PAUP using maximum likelihood, neighbor-joining, maximum parsimony, and RAxML using maximum likelihood).

The DNA extraction and PCR amplification were conducted using the protocols of Darienko et al. [21]. The alignment and phylogenetic analyses were conducted using the methodology published by Darienko et al. [9]. The SSU rDNA sequences were included in a large data set of 67 taxa of representatives belonging to the Ulvophyceae s.str. with 1780 unambiguously aligned positions. The alignment was conducted according to the secondary structure of SAG 9.90 *Chlorocystis cohnii* (see Supplemental Figure S1 in Darienko et al. [9]). To obtain a higher resolution, a concatenated data set of SSU and ITS consisting of 36 taxa with 2389 unambiguously aligned positions was created. We used the Automated Model Selection tool implemented in PAUP* version 4.0a (build 169; [22]) for the decision about which evolutionary model best fitted both data sets. The settings of the best models are given in the legends of Figure 1 and Figure 2. EMBL/GenBank accession numbers of published sequences and strain designations are provided in both of these figures. Phylogenetic trees were constructed using distance, parsimony, and maximum likelihood criteria using PAUP [22], and the robustness of the tree topologies was proven with different Bayesian and bootstrap analyses (1000 replicates). In addition, the programs RAxML version 8.2.12 [23], MrBayes version 3.2.7a [24], and PHASE package 2.0 [25,26,27,28,29] were used.

The secondary structures of the ITS-2 sequences were folded according to the approach introduced by Darienko and Pröschold [30] for non-marine ulvophytes. They used the computer programs mfold [31]. For an overview of the distribution, we used the V4 region of the SSU rDNA sequences to search entries in GenBank. For V4 the following approach was used for the BLAST N searches: 100% coverage, 100% identity [32]. The geographical coordinates of each coccoid and sarcinoid ulvophytes are summarized in the Supplemental Material (Table S1). The same records were found using the V9 region despite the fact that this region is not species-specific like the V4 region. Therefore, we used V4 to construct the haplotype networks, and we used the TCS network and geographical mapping tools [33,34] implemented in PopART [35] for all genera.

## 3. Results and Discussion

Darienko et al. [9] have already demonstrated that marine coccoid green algae are members of different lineages among the Ulvophyceae. In their study, the strain MBIC 10461 had no affiliation to an order. The strain SAG 2662 together with this strain formed a new lineage within the ulvophytes, which is highly supported by all of the bootstrap and Bayesian analyses (Figure 1 and Figure 2). As a consequence of our phylogenetic analyses, we propose a new genus: *Solotvynia* (described below). The highly supported tree topology shown in Figure 1 was tested using approximately unbiased tests of the user-defined trees implemented in PAUP to find out if the genus *Solotvynia* also represents a new order within the Ulvophyceae. All of these trees (Tree 2: *Solotvynia* sister of *Chlorocystis*; Tree 3: *Solotvynia* sister of *Sykidion*; Tree 4: *Solotvynia* sister of Ignatiales; Trees 5–10: collapse of the common branches 1–6 marked in Figure 1) were significantly worse (*p*-values < 0.05) compared to the best tree (Tree 1; Figure 1), which confirms that the genus *Solotvynia* represents a new order among the Ulvophyceae (described below). A detailed analysis of the intraspecific, interspecific, and intergeneric variations as well as the genetic synapomorphies of the orders is the topic of a separate publication (Pröschold and Darienko, in prep.) The SSU sequence of strain CCMP 1293 is almost identical (99.7%) to that of *Symbiochlorum hainanense* (MH061387). The sequences of the two strains NIES-1838 and NIES-1839 were included in the concatenated dataset of SSU and ITS, which were aligned according to their secondary structures. The phylogenetic analyses demonstrate that both strains belong to *Chlorocystis john-westii*. However, they were not identical to each other or to the other existing reports of this species (Figure 2). All strains belonging to this species showed 1.13% variability in their SSU sequences, but no differences were detected in the ITS-2 barcode, as demonstrated in Darienko et al. [9]. Both strains NIES-1838 and NIES-1839 showed little variations in the ITS-2 secondary structure, but the ITS-2 barcodes were identical (barcode C6 in Darienko et al. [9]). To summarize, the newly investigated strains belong to the orders Ignatiales, Chlorocystidales, and Solotvyniales ordo nov. (described below).

The use of the ITS-2/CBC approach for species delineation for the genera *Desmochloris*, *Chlorocystis,* and *Sykidion* had already been demonstrated by Darienko et al. [9]. Therefore, we compared the ITS-2 secondary structure of the strains SAG 2662, CCMP 1293, and UTEX 2012. All strains showed the typical ITS-2 structure for ulvophytes (Helices I–III, IV is missing).

**Figure 3:**
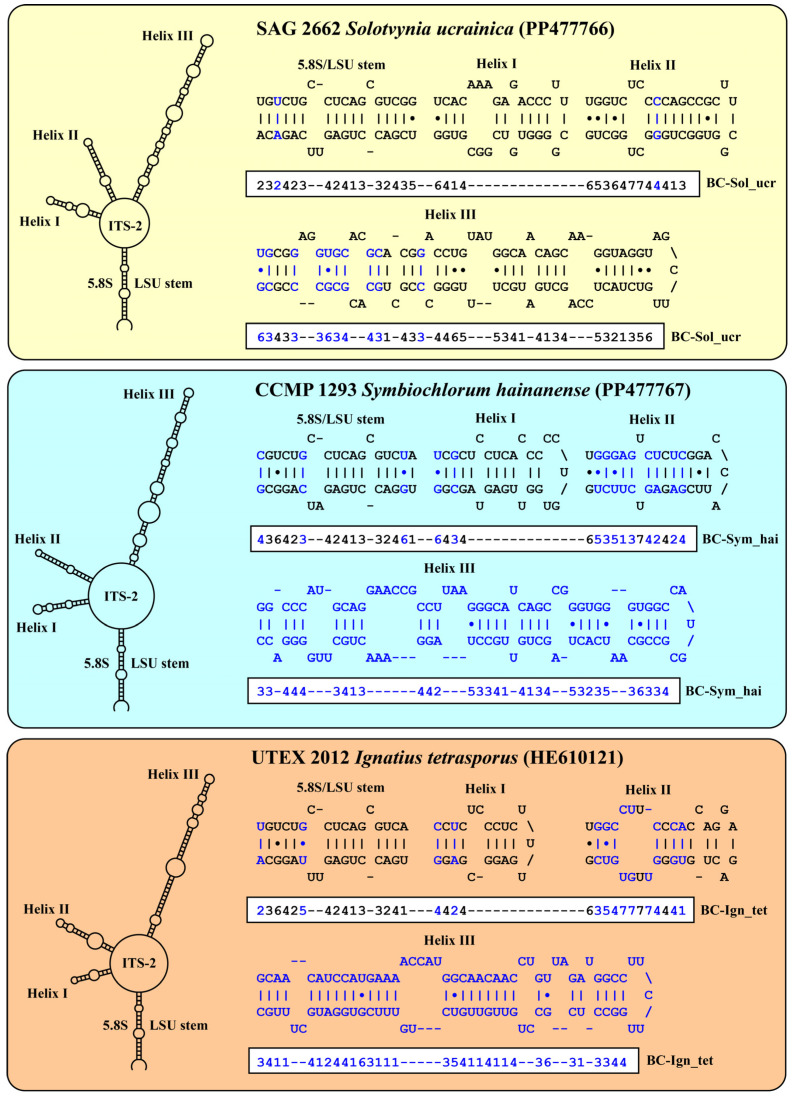
Comparison of the conserved region of ITS-2 among the species of *Solotvynia*, *Symbiochlorum*, and *Ignatius*. Extraction of this region and translation into a number code for its usage as barcode. Number code for each base pair: 1 = A–U; 2 = U–A; 3 = G-C; 4 = C–G; 5 = G·U; 6 = U·G; 7 = mismatch. Compensatory base changes (CBCs/HCBCs) are highlighted in blue.

The structures and ITS-2 barcodes delineated using the conserved region of ITS-2 are given in Figure 3. The barcodes differ from those of the other coccoid members of the ulvophytes and we found compensatory base changes in the conserved region of ITS-2. A comparison of the *Solotvynia* barcode to the species of *Desmochloris*, *Chlorocystis,* and *Sykidion* highlighted eleven CBCs/HCBCs (highlighted in blue in Figure 3). The ITS-2 sequences of *Symbiochlorum hainanense* and *Ignatius tetrasporus* differ in many bases, particularly Helix III.

**Figure 4:**
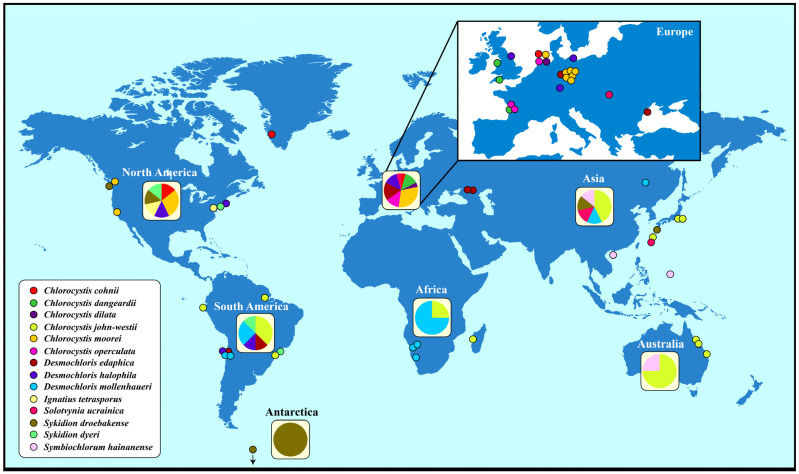
Distribution of the coccoid and sarcinoid green algae belonging to the Ulvophyceae around the world. The geographical origin of *Sykidion marina* is unknown.

To gain an overview of the geographical distribution of coccoid and sarcinoid ulvophytes, we used the haplotype network approach implemented within PopART by adding the geographical origin of the strains (Table S1). Generally, these ulvophytes have a worldwide distribution (Figure 4). However, certain species such as *Chlorocystis john-westii* and *Desmochloris mollenhaueri* were mostly reported from the Southern Hemisphere whereas *Chlorocystis moorei* and *Desmochloris halophila* were only found in the Northern Hemisphere. *Sykidion droebakense* is the only species that was found in Antarctica, but this is probably caused by a lack of investigations. Most records of coccoid and sarcinoid ulvophytes were reported from Europe and North America. Some species were only found once or twice (*Chlorocystis cohnii*, *C. dilatata*, *Solotvynia ucrainica*, *Ignatius tetrasporus,* or *Symbiochlorum hainanense*).

The geographical distribution is much wider if you check the records in the GBIF database (https://www.gbif.org; accessed on 3 April 2024). More than 1000 records of the genera *Desmochloris*, *Chlorocystis*, *Solotvynia*, *Sykidion*, *Ignatius*, and *Symbiochlorum* are documented and confirm that all genera have a worldwide distribution (Table S2). Unfortunately, most of the records cannot be proven and can only be identified at the generic level. Only the SSU entries used for Figure 4 could be identified at the species level. Several entries in the GBIF are questionable. For example, 75 records of *Ignatius* were reported in the GBIF, most of them from marine habitats around the world, but only the authentic strain isolated from soil is available and no other isolate has been studied so far. Therefore, the GBIF distribution pattern of *Ignatius tetrasporus* has to be taken with caution. Careful considerations should be also made for the other genera.

A comparison of the morphology showed similarities, but also differences, among the investigated strains. NIES-1838 and NIES-1839 are morphologically identical to the other strains of *Chlorocystis john-westii*, as already indicated by the phylogenetic analyses. 

**Figure 5:**
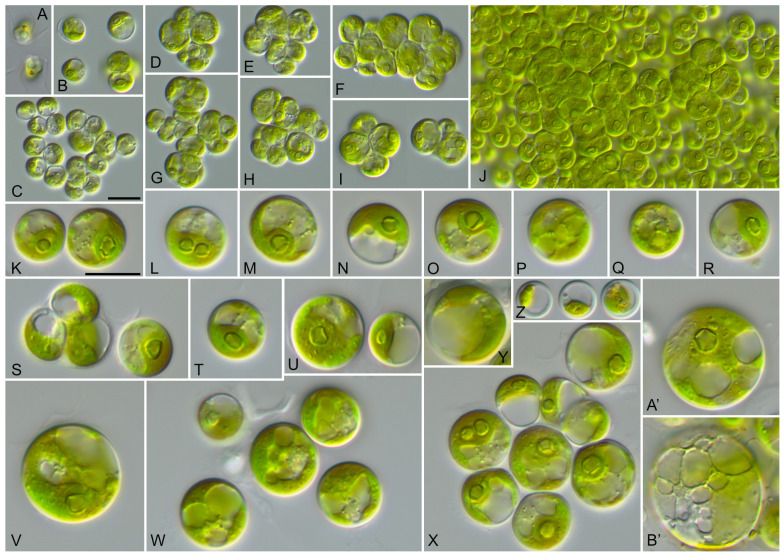
Morphology and phenotypic plasticity of *Solotvynia ucrainica* (SAG 2662) grown on SWES medium. (**A**). Quadriflagellated zoospores; (**B**,**C**). Young vegetative cells; (**D**–**I**). Tetrad formation; (**J**). Parenchyma-like crust; (**K**–**N**). Vegetative cells with cup-shaped chloroplasts; (**O**–**X**). Mature cells of different ages with reticulated chloroplasts; (**Y**–**B′**). Old cells with reticulated chloroplasts and numerous vacuoles. Scale bar = 10 µm.

**Figure 6:**
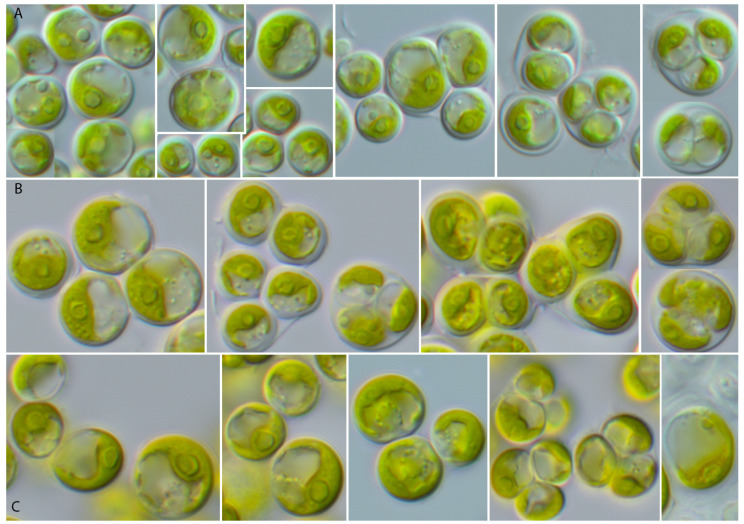
Morphology and phenotypic plasticity of *Solotvynia ucrainica* (SAG 2662) grown on different media. (**A**). ES medium (freshwater); (**B**). 1/2SWES medium (brackish); (**C**). *Dunaliella* medium (hypersaline). Scale bar = 10 µm.

The vegetative cells of SAG 2662 showed a spherical morphology at all ages (Figure 5). They form tetrads, which stick together to form a parenchyma-like crust (Figure 5J). The chloroplasts are cup-shaped (Figure 5K–N) similar to *Chlorocystis* and become reticulated with age (Figure 5O–B’). The chloroplasts contain single or sometimes two pyrenoid(s) (Figure 5L). The zoospores are quadriflagellated (Figure 5A). As shown in Figure 1, this strain is similar to the strain MBIC 10461 in our analysis. Unfortunately, the morphology of that strain cannot be compared because the strain is currently not available in public culture collections. The ecology of both strains differs. SAG 2662 originated from a hypersaline lake, whereas the strain MBIC 10461 was isolated from the open sea. To test if the strain SAG 2662 can survive in other conditions, we cultivated it on freshwater, brackish, and hypersaline media (see Section 2). In our experiment, which lasted for four weeks, the strain survived all conditions and showed only minor morphological variations (Figure 6).

**Figure 7:**
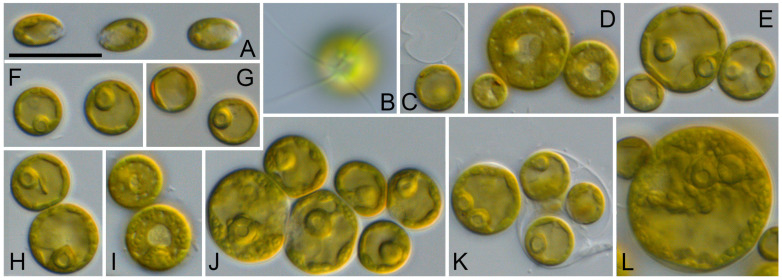
Morphology and phenotypic plasticity of *Symbiochlorum hainanense* (CCMP 1293) grown on SWES medium. (**A**). Zoospores shortly after settlement; (**B**). Quadirflagellated zoospore; (**C**,**F**,**G**). Young cells shortly after settlement with eye spot and empty sporangium cell wall; (**D**,**E**,**H**,**I**). Vegetative cells in apical and middle sections showing the structure of chloroplast; (**J**–**L**). Vegetative cells of different age. Scale bar = 10 µm.

As shown above, the SSU and ITS sequences of the CCMP 1293 strain were almost identical to those of *Symbiochlorum hainanense*. However, the morphology presented in Figure 7 shows differences to the original description of this species published by Gong et al. [13]. They observed spherical cells with a size of 5–12 µm, multiple chloroplasts with pyrenoids, and asexual reproduction through aplanospores. In contrast, we found larger cells (Figure 7J–L) with perforated single cup-shaped chloroplasts with one or two pyrenoid(s) surrounded by large starch grains. The asexual reproduction occurs through quadriflagellated zoospores. Considering our results, we emend the original descriptions of the genus and species (see below).

### Taxonomic Revisions and Diagnoses

As demonstrated above, the newly investigated strains belong to three different genera. The strains NIES-1838 and NIES-1839 represent new isolates of *Chlorocystis john-westii*, which has been confirmed by molecular analyses and morphological observations. The strain SAG 2662 (= ULVO-129) represents a new genus and order as demonstrated by phylogenetic analyses, its ITS-2 secondary structure, and its morphology (for a comparison to other coccoid and sarcinoid genera, see Table S3). The strain CCMP-1293 is almost identical in terms of its SSU and ITS rDNA sequences to *Symbiochlorum hainanense*. However, it differs in its morphology and reproduction from the original description by Gong et al. [13]. As a consequence of our study, we formally revise this generic and species description and propose the following new order and genus.

**Solotvyniales** ordo nov.
*Description*: Chlorophyta unicellular. Chloroplast cup-shaped, parietal or reticulate, with pyrenoid. Asexual reproduction by quadrilagellated zoospores.
*Type family* (designated here): Solotvyniaceae fam. nov.
*Description*: Characters as for the order.
*Type genus* (designated here): *Solotvynia* gen. nov.
***Solotvynia*** gen. nov.
*Description*: Vegetative cells are solitary, spherical, slightly flattened or polygonal, and form tetrads. Cells are uninucleated with cup-shaped or saucer-shaped chloroplasts containing a pyrenoid surrounded by several large starch grains. Chloroplasts become irregularly reticulated with age. The cell wall is thin in young cells and becomes slightly thicker (around 1 µm) in old cells. Vegetative cells possess one up to several large vacuoles occupying around half of cell volume. Reproduction by aplano- or zoospores. Release of zoospores is apical, with one side of the sporangia rupturing. Zoospores are released in a mucilage envelope, and disappear after several minutes following liberation. Zoospores do not have cell walls or scales. Zoospores are quadriflagellated with a counterclockwise basal body orientation. Sexual reproduction was not observed.
*Diagnosis*: Differs from other coccoid genera of the Ulvophyceae by SSU and ITS rDNA sequences.
*Type species* (designated here): *Solotvynia ucrainica* sp. nov.
***Solotvynia ucrainica*** sp. nov. (Figure 5).
*Description*: Young vegetative cells are solitary or form spherical tetrads. Tetrads stick together and form a crust-like biofilm without mucilage. Young cells are 5.2 up to 7.4 µm in diameter. Chloroplasts are parietal lobated, with a single pyrenoid surrounded by several starch grains. Mature vegetative cells are spherical and 8.2–12.9 µm in diameter. Chloroplasts are parietal lobated containing one or several pyrenoid(s) surrounded by several starch grains. Cells with one or several vacuole(s) are located in the cytoplasm. The cell wall is 0.5–0.8 µm thick. Old cells that are 7.4–12.4 (–18) µm in diameter contain a large vacuole compressing the chloroplast to the cell wall. The chloroplast shape is then not recognizable. The cell wall becomes thicker, up to 1.2 µm. Old cells are often surrounded by exfoliated cell walls, which probably originated from the sporangial walls. The one-year-old culture remained green in color. Sexual reproduction was not observed. Asexual reproduction occurred through quadriflagellated zoospores with flagella of equal length. Zoosporangia are 10.2–12.2 µm in diameter and contain four daughter cells. The release of the zoospores occurred through an apical circular pore. Zoospores are 6.0–7.0 µm long × 3.5–4.0 µm wide, with parietal chloroplasts and pyrenoid and anterior-lateral stigma. The zoospores are released in a mucilage vesicle, which dissolves in several minutes. After 10–20 min active movement, the zoospores settle down and become spherical and 5.5–6.5 µm in diameter. Young cells retain the stigma for some time.
*Diagnosis*: SSU-ITS sequences (GenBank: PP477766) and ITS-2 Barcode: Sol_ucr in Figure 3.
*Holotype* (designated here): The authentic strain SAG 2662 (= ULVO-129) cryopreserved in a metabolically inactive state at the Culture Collection of Algae (SAG), University of Göttingen, Germany.
*Type locality*: Ukraine, Solotvyno, from a hypersaline artificial lake (47°57′20″ N, 23°52′16″ E).
*Etymology*: The species is named after the origin of the authentic strain.
*Comment*: The strain MBIC 10461 could represent another species of *Solotvynia*, but this cannot be confirmed at this stage because no ITS rDNA sequences or morphological data are available. We could not obtain this strain for further investigation.
The strain SAG 2662 was isolated from a sample which was dominated by *Dunaliella pseudosalina* Massjuk.
***Symbiochlorum*** S.Q. Gong & Z.Y. Li emend.
*Emended description*: Young cells are broadly ellipsoidal up to spherical. The chloroplasts form a hollow sphere with small perforations, and contains one pyrenoid; mature cells become net-like and contain several pyrenoids. The cells uninucleate. The cell wall is relatively thin and becomes thicker with age. Reproduction occurs through zoospores and aplanospores. Zoospores are quadriflagellated with anterior stigma. The chloroplasts of the zoospores are parietal and slightly perforated, with one pyrenoid. The zoospores are surrounded by thin cell walls and become spherical after moving.
Type species: *Symbiochlorum hainanense* S.Q. Gong & Z.Y. Li emend.
***Symbiochlorum hainanense*** S.Q. Gong & Z.Y. Li emend. (Figure 7).
*Emended description*: Young cells are broadly ellipsoidal up to spherical and 5.4–8.8 × 4.0–7.7 µm in diameter. Their chloroplasts are very special. At the beginning, the chloroplasts form a hollow sphere with an apical opening with small perforations, containing one pyrenoid. After the cells are 7.0 µm in diameter, they start to form net-like chloroplasts and possess 2–3 pyrenoids, but the hollow sphere can still be observed. The chloroplasts of the mature cells are net-like with invaginations, where the pyrenoid is often located. Mature cells can contain several (from 2 up to 7) pyrenoids. Pyrenoids are well visible and are surrounded by large starch grains. The mature cells are spherical and 14.5 up to 27 µm in diameter or broadly ellipsoidal at 13.9–27.5 × 12.4–19.3 µm in size, and they uninucleate. The cell wall is relatively thin, around 0.4–0.5 µm thick. The color of the chloroplasts is brownish. Old cells are spherical up to 26.0–27.0 µm in diameter. The cell wall becomes up to 1.3 µm thick. Reproduction occurs through zoospores and aplanospores. Zoosporangia can contain 2–8 cells. Zoosporangia are spherical or broadly ellipsoidal and are 13.4–16.0 × 12.4–15.9 µm in size. Spores are released through the rupture of the sporangial cell wall. Zoospores are quadriflagellated and 5.9–6.1 × 3.5–3.7 µm in size. The flagella are 1.5–2.0 × longer than the body of zoospores. The stigma is anterior and the cells are unnucleated. The chloroplasts are parietal, slightly perforated, and have one pyrenoid. The zoospores are surrounded by thin cell walls and become spherical after moving.
*Emended diagnosis*: SSU-ITS sequences (GenBank: PP477767) and ITS-2 Barcode: Sym_hai in Figure 3.
*Epitype* (designated here): The strain CCMP 1293 has been cryopreserved in a metabolically inactive state at the Provasoli-Guillard National Center for Culture of Marine Phytoplankton, Bigelow, Maine, USA.

## Data Availability

Data are contained within the article and Supplementary Materials.

## Supplementary Materials

The following are available online at https://www.mdpi.com/article/10.3390/microorganisms12050868/s1, Table S1. GenBank entries (accession numbers) found by BLAST N search (100% coverage; 100% identity) using the V4 region of the SSU rDNA of each species. For each entry the geographical origin and the habitat are given; Table S2. Distribution of the investigated coccoid and sarcinoid genera found in the GBIF database (https://www.gbif.org; accessed on 3 April 2024); Table S3. Morphology of coccoid and sarcinoid genera belonging to the Ulvophyceae.
