# Supplementary material for: Solotvynia, a New Coccoid Lineage among the Ulvophyceae (Chlorophyta)
Source: Microorganisms. 2024 Apr 26;12(5):868. doi: 10.3390/microorganisms12050868 (PMC11123690; doi:10.3390/microorganisms12050868)
Supplement: Supplementary file 1 [file microorganisms-12-00868-s001.zip › Table_S1.pdf]

Table S1: GenBank entries (accession numbers) found by BLAST N search (100% coverage; 100% identity) using the V4 region of the SSU rDNA of each species. To each entry the geographical origin and after the habitat are given.

species	strain	accession	Habitat	continent	coordinates
Desmochloris halophila	CCAP 6006/1	FM882216	marine	North America (USA, MA)	41.392045 N, -70.620814 E
	CCAP 6006/4	MW714125	soil	South America	-27.066667 N, -70.816667 E
	RCC 2945	KT860927	marine	Europe, North Sea	54.000000 N, 0.000000 E
	SAG 2565	MH703754	soil	West Europe	54.416667 N, 13.366667 E
	SAG 2397	MW714126	freshwater	West Europe	49.385833 N, 11.471944 E
Desmochloris edaphica	CCAP 6006/5	MW714127	soil	East Europe	45.254722 N, 30.203333 E
	CCAP 6006/6	MW714128	soil	South America	-27.066667 N, -70.816667 E
	TT-4-1	MT968399	soil, saline	West Europe	51.467324 N, 11.770714 E
	ACSSI 181	MH102328	soil	East Europe	47.889167 N, 44.013889 E
	ACSSI 180	KY086483	soil	East Europe	47.889167 N, 44.013889 E
Desmochloris mollenhaueri	CCAP 6006/2	FM882217	soil	Africa	-31.616667 N, 18.716667 E
	CCAP 6006/3	FM882218	soil	Africa	-28.612340 N, 16.654331 E
	CCAP 6006/7	MW714129	soil	Africa	-28.226646 N, 17.025668 E
	CCAP 6006/8	MW714130	soil	South America	-27.066667 N, -70.816667 E
	CCAP 6006/9	MW714131	soil	South America	-27.066667 N, -70.816667 E
	JB13	KF791549	soil saline	Asia China	46.585352 N, 125.161214 E
Chlorocystis cohnii	SAG 9.90	MW714132	marine	West Europe	54.182061 N, 7.887631 E
	SCCAP K-0421	MW714133	marine, endophyte	North America, Greenland	64.434722 N, -50.269444 E
Chlorocystis dangeardii	SAG 8.86	MW714140	marine	West Europe	53.277121 N, -3.807850 E
	CCAP 211/25	MW714141	brackish	West Europe	50.358333 N, -4.166667 E
	CCAP 233/1	MW714142	marine	West Europe	45.513149 N, -1.122879 E
Chlorocystis john-westii	CCAP 6005/4	MW714146	marine, endophyte	South America	-23.693247 N, -45.461417 E
	CCAP 6005/5	MW714147	marine, endophyte	Australia	-23.853814 N, 151.278133 E
	CCAP 6005/10	MW714143	marine, endophyte	South America	-3.558611 N, -80.519142 E
	CCAP 6005/11	MW714144	marine, endophyte	Australia	-19.418678 N, 147.233276 E
	CCAP 6005/12	MW714145	marine, endophyte	Australia	-19.418678 N, 147.233276 E
	UTEX 2846	MW714148	marine, endophyte	South America	-5.208550 N, -45.393026 E
	NIES-1838	PP477768	marine	Asia	35.649862 N, 139.825201 E
	NIES-1839	PP477769	marine	Asia	35.649862 N, 139.825201 E
	CCAP 6005/13	MK541803	marine	Africa	-20.736302 N, 44.002005 E
	MBIC 10446	AB058345	marine	Asia	26.570775 N, 128.02559 E
Chlorocystis operculatum	SAG 19.92	MW714134	marine	West Europe	45.513149 N, -1.122879 E
	CCMP 435	MW714135	marine	West Europe	45.513149 N, -1.122879 E
	SAG 11.90	MW714136	marine	West Europe	54.182061 N, 7.887631 E
Chlorocystis dilatatum	SAG 12.90	MW714139	marine	West Europe	54.182061 N, 7.887631 E
Chlorocystis moorei	CCAP 6005/6	MW714137	marine	West Europe	54.182061 N, 7.887631 E
	CCMP 2288	MW714138	marine	North America (USA, Washington)	48.535278 N, -123.031111 E
	TT-4-1-D	MT968404	soil, saline	West Europe	51.467324 N, 11.770714 E
	TT-4-1-O	MT968406	soil, saline	West Europe	51.467324 N, 11.770714 E
	TT-4-1-M	MT968405	soil, saline	West Europe	51.467324 N, 11.770714 E
	NN-4-1-T	MT968403	soil, saline	West Europe	51.467324 N, 11.770714 E
	NN-4-1-S	MT968402	soil, saline	West Europe	51.467324 N, 11.770714 E
	NN-4-1-Q	MT968401	soil, saline	West Europe	51.467324 N, 11.770714 E
	voucher	DQ821520	marine	North America (USA, California)	38.755113 N, -121.353309 E
Sykidion marinum	UTEX 1445	MW714149	unknown	unknown	unknown
Sykidion dyeri	CCMP 257	MW714150	marine	North America (USA, CT)	41.216667 N, -73.066667 E
	BE-001	MK457458	marine	South America (Brasil)	-22.892939 N, -43.124084 E
Sykidion droebakense	CCMP 258	MW714151	marine	North America (British Columbia)	48.407326 N, -123.980713 E
	CCMP 438	MW714152	marine	Antarctica	-64.774167 N, -64.052222 E
	NKY372003	LC505539	marine	Asia	30.347925 N, 130.524483 E
Solotvynia ucrainica	SAG 2662	PP477766	saline	West Europe	47.955556 N, 23.871111 E
	MBIC 10461	AB058346	marine	Asia	26.546766 N, 128.055267 E
Symbiochlorum hainanensis	CCMP 1293	PP477767	marine	Asia	7.540278 N, 134.589167 E
	CCTCC M2018096	MH061387	marine	Asia	18.208480 N, 109.401855 E
Ignatius tetrasporus	UTEX 2012	FN562432	soil	North America (USA, Pennsylvania)	41.396201 N, -75.196287 E
