# Supplementary material for: Solotvynia, a New Coccoid Lineage among the Ulvophyceae (Chlorophyta)
Source: Microorganisms. 2024 Apr 26;12(5):868. doi: 10.3390/microorganisms12050868 (PMC11123690; doi:10.3390/microorganisms12050868)
Supplement: Supplementary file 1 [file microorganisms-12-00868-s001.zip › Table_S2.pdf]

**Table S2**: Distribution of the investigated coccoid and sarcinoid genera found in the GBIF database (https://www.gbif.org)

| Genus | # of records | Sources |
|---|---|---|
| ***Desmochloris*** | **484** | https://www.gbif.org/species/2652025 |
| ***Chlorocystis* (syn. *Halochlorococcum*)** | **52** | https://www.gbif.org/species/2646192 |
| | **311** | https://www.gbif.org/species/9798860 |
| ***Solotvynia*** | **2** | this study |
| ***Sykidion* (syn. *Pseudoneochloris*)** | **25** | https://www.gbif.org/species/2642314 |
| | **77** | https://www.gbif.org/species/2646252 |
| ***Ignatius*** | **75** | https://www.gbif.org/species/2640526 |
| ***Symbiochlorum*** | **2** | https://www.gbif.org/species/10620661, this study |
| **Total** | **1028** | |
