# Supplementary material for: Solotvynia, a New Coccoid Lineage among the Ulvophyceae (Chlorophyta)
Source: Microorganisms. 2024 Apr 26;12(5):868. doi: 10.3390/microorganisms12050868 (PMC11123690; doi:10.3390/microorganisms12050868)
Supplement: Supplementary file 1 [file microorganisms-12-00868-s001.zip › Table_S3.pdf]

Table S3: Morphology of coccoid and sarcinoid genera belonging to the Ulvophyceae.

	Solotvynia	***Desmochloris***	***Chlorocystis***	***Halochlorococcum***	***Sykidion***	***Ignatius***	***Symbiochlorum***	***Symbiosphaera***
**cell shape**	spherical forming tetrads	broadly ellipsoidal - spherical forming packages of 2-8 cells	spherical - subspherical	spherical - subspherical	spherical - slightly flattened	spherical - saccate ovoidal	broadly ellipsoidal - spherical	spherical
**cell size**	8-13 µm	6-12 µm	12-25 (-40) µm	30-40 (-50) µm	8-19 µm	12-25 µm	14-27 µm	12-25 µm
**nucleus**	1	1	1	1	1	1	1	1
**chloroplast**	parietal lobated	cup-shaped with incisions	reticulated	reticulated	cup-shaped	parietal lobated	hollow sphere with perforations	reticulated
**pyrenoid**	1(- several)	1	1	1	1	1(- several)	2-7	1
**zoospores**	+	+	+	+	+	+	+/-	+
**number of flagella**	4	2	4	4	2	4	4	4
**number of species**	1	3	6	1	3	1	1	1
***Codiolum*-stage**	-	-	+ (*C. cohnii*)	+	-	-	-	-
**phylogenetic status**	resolved	resolved	resolved	unresolved	resolved	resolved	resolved	unresolved
**reference**	this study	Darienko *et al.* [9]	Kornmann & Sahling [6] Darienko *et al.* [9]	Kornmann & Sahling [6] Dangeard [7]	Darienko *et al.* [9]	Bold & MacEntree [10]	this study Gong *et al.* [13]	Moewus [5]
